# Supplementary material for: Molecular basis of cullin-3 (Cul3) ubiquitin ligase subversion by vaccinia virus protein A55
Source: J Biol Chem. 2019 Feb 28;294(16):6416–29. doi: 10.1074/jbc.RA118.006561 (PMC6484134; doi:10.1074/jbc.RA118.006561)
Supplement: Supporting Information [file supp_294_16_6416__index.html]

Molecular basis of cullin-3 (Cul3) ubiquitin ligase subversion by vaccinia virus protein A55 — Structure of Cul3 in complex with vaccinia virus protein A55 — Molecular basis of cullin-3 (Cul3) ubiquitin ligase subversion by vaccinia virus protein A55 — Structure of Cul3 in complex with vaccinia virus protein A55 — Supporting Information 

# Molecular basis of cullin-3 (Cul3) ubiquitin ligase subversion by vaccinia virus protein A55

## Supporting Information

- Supporting Information (to be published online) - Supplementary Figures 1-7
