# Supplementary material for: Molecular basis of cullin-3 (Cul3) ubiquitin ligase subversion by vaccinia virus protein A55
Source: J Biol Chem. 2019 Feb 28;294(16):6416–29. doi: 10.1074/jbc.RA118.006561 (PMC6484134; doi:10.1074/jbc.RA118.006561)
Supplement: Supporting Information [file supp_RA118.006561_141719_2_supp_292179_pnjg25.pdf]

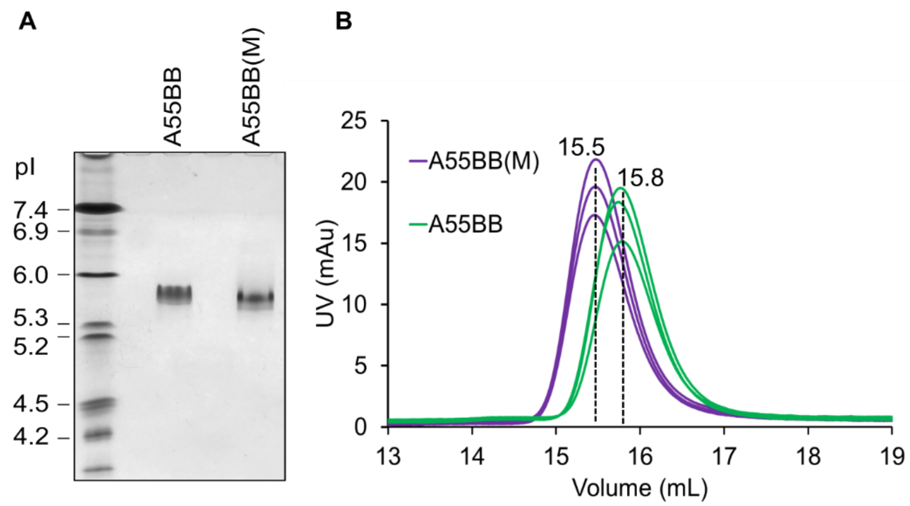

Figure S1. Validation of A55 reductive methylation (M) used for crystallization of the A55BB/Cul3NΔ22 complex. A. Isoelectric focusing gel analysis of unmethylated A55BB and methylated A55BB(M) showing a drop in the isoelectric point (pI) of A55BB(M) as a result of lysine methylation. **B.** Analytical SEC analysis showing different elution times of the methylated (purple) and unmethylated (green) A55BB (1). The distinct elution volumes in millilitre are shown above the peaks. Experiments were performed in triplicate.

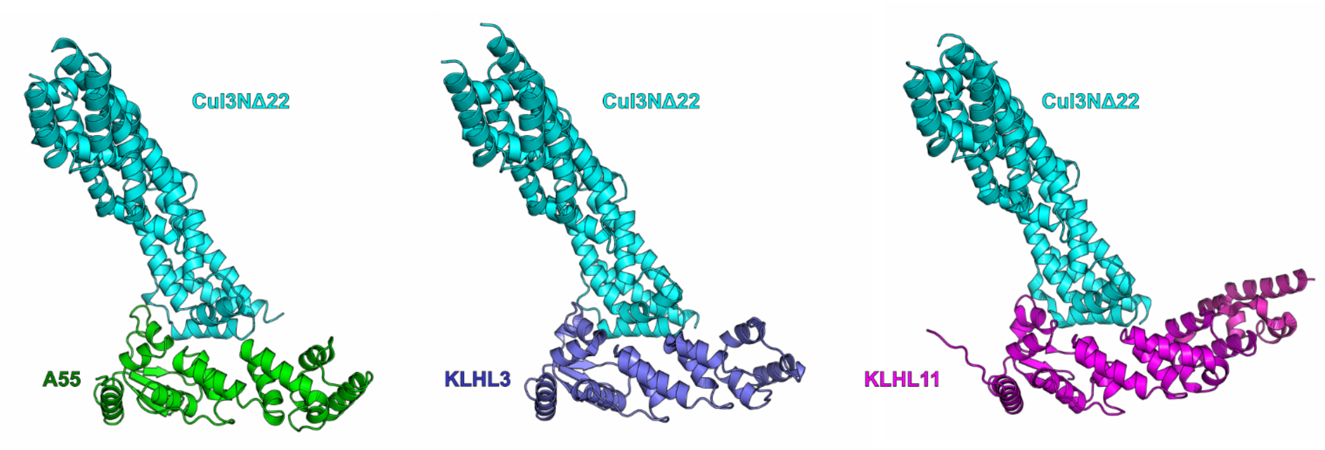

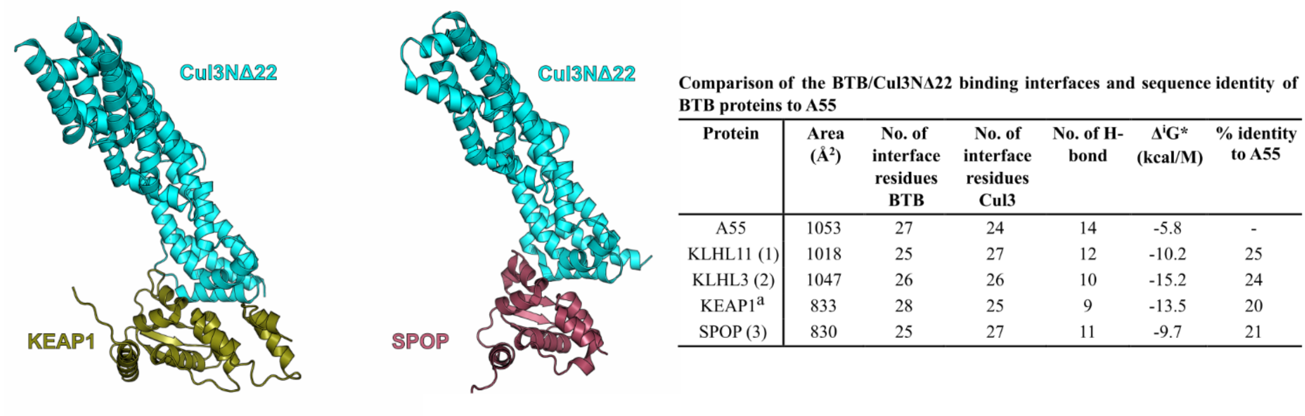

Comparison of the BTB/Cul3NΔ22 binding interfaces and sequence identity of BTB proteins to A55

Protein	Area (Å^2)	No. of interface residues BTB	No. of interface residues Cul3	No. of H-bond	Δ^iG* (kcal/M)	% identity to A55
A55	1053	27	24	14	-5.8	-
KLHL11 (1)	1018	25	27	12	-10.2	25
KLHL3 (2)	1047	26	26	10	-15.2	24
KEAP1[a]	833	28	25	9	-13.5	20
SPOP (3)	830	25	27	11	-9.7	21

Figure S2. Comparison of five BTB/Cul3 complex structures. The structures of A55 (green), KLHL3 (purple), KLHL11 (magenta), KEAP1 (gold) or SPOP (pink) in complex with Cul3 (cyan) are shown as ribbon diagrams. Inset table summarizes the interface areas, number of interface residues from the BTB proteins and from Cul3, number of hydrogen bonds, solvation free energy gain (Δ^iG, a measure of contribution from hydrophobic interaction, where the more negative the value is, the bigger the contribution) as calculated by PDBePISA (2), and percentage sequence identity to A55. The PDB accession codes for KLHL11, KLHL3, KEAP1 and SPOP are 4APF, 4HXI, 5NLB and 4EOZ, respectively (3-5).

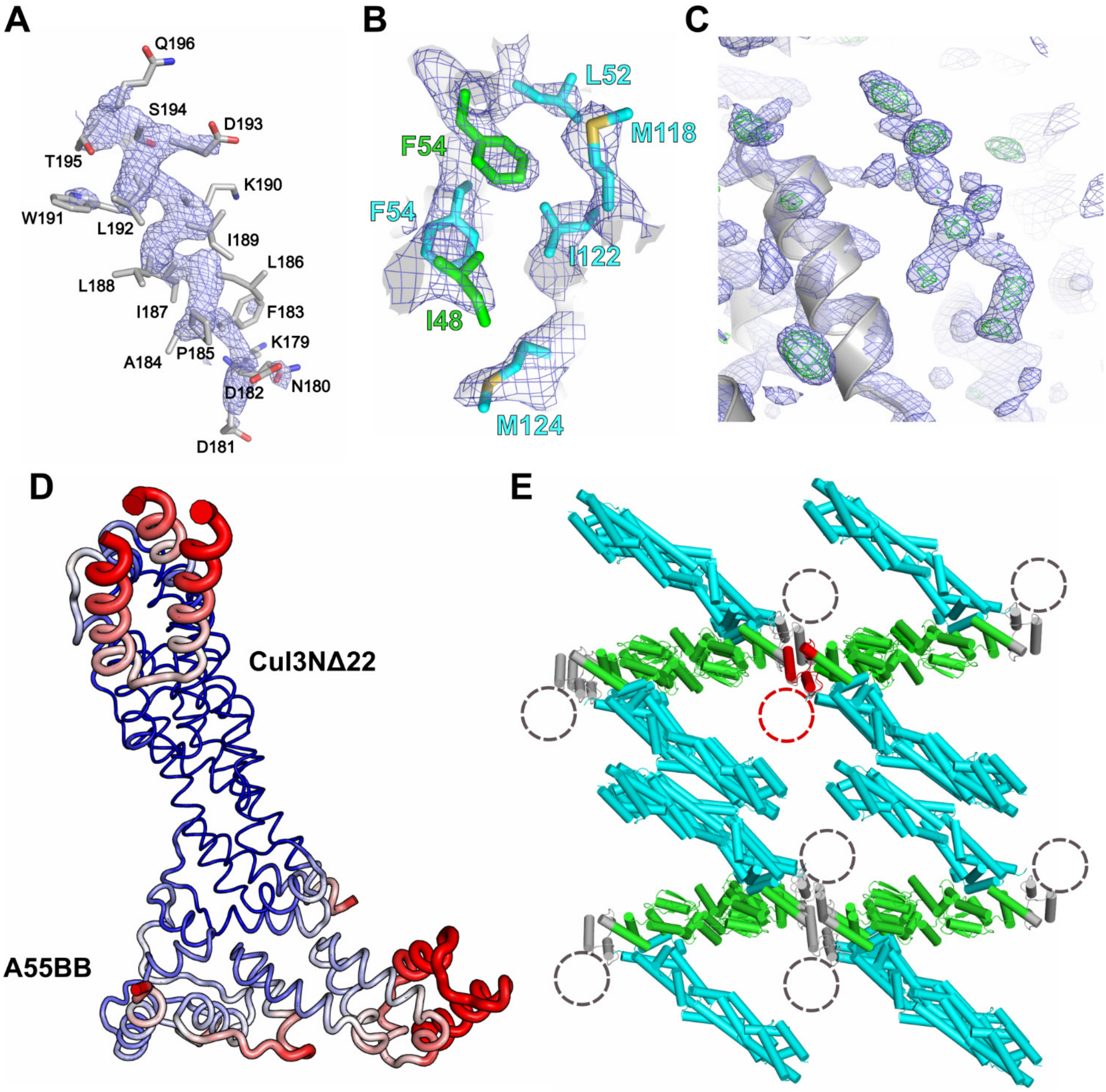

Figure S3. Quality assessment of the structure of the BACK domain. A. The last modelled helix of the A55 BACK domain (residues 180-196) is shown as side chain sticks, with corresponding $2F_O$-F_C map (1.5σ) shown as mesh. **B.** The $2F_O$-F_C map (1.5σ) for the well-resolved hydrophobic interactions at the A55 (green)/Cul3 (blue) interface is shown as mesh. **C**. Electron densities beyond the last helix of the A55 BACK domain are evident in the $2F_O$-F_C (purple mesh, 1.5σ) and F_O-F_C (green mesh, 3.5σ) maps. **D.** The A55BB/Cul3NΔ22 structure is shown as tubes where the tube width and color varies from thin and blue (low B-factor) to fat and red (high B-factor). **E.** Crystal lattice showing the packing of the A55/Cul3 complex. Helices are shown as tubes. Cul3 and A55 are colored in cyan and green, respectively. The BACK domain of a selected A55 molecule is colored in red, while the BACK domains of the symmetry-related molecules are colored in grey. Regions showing discontinuous density for the remainder of the BACK domain are circled.

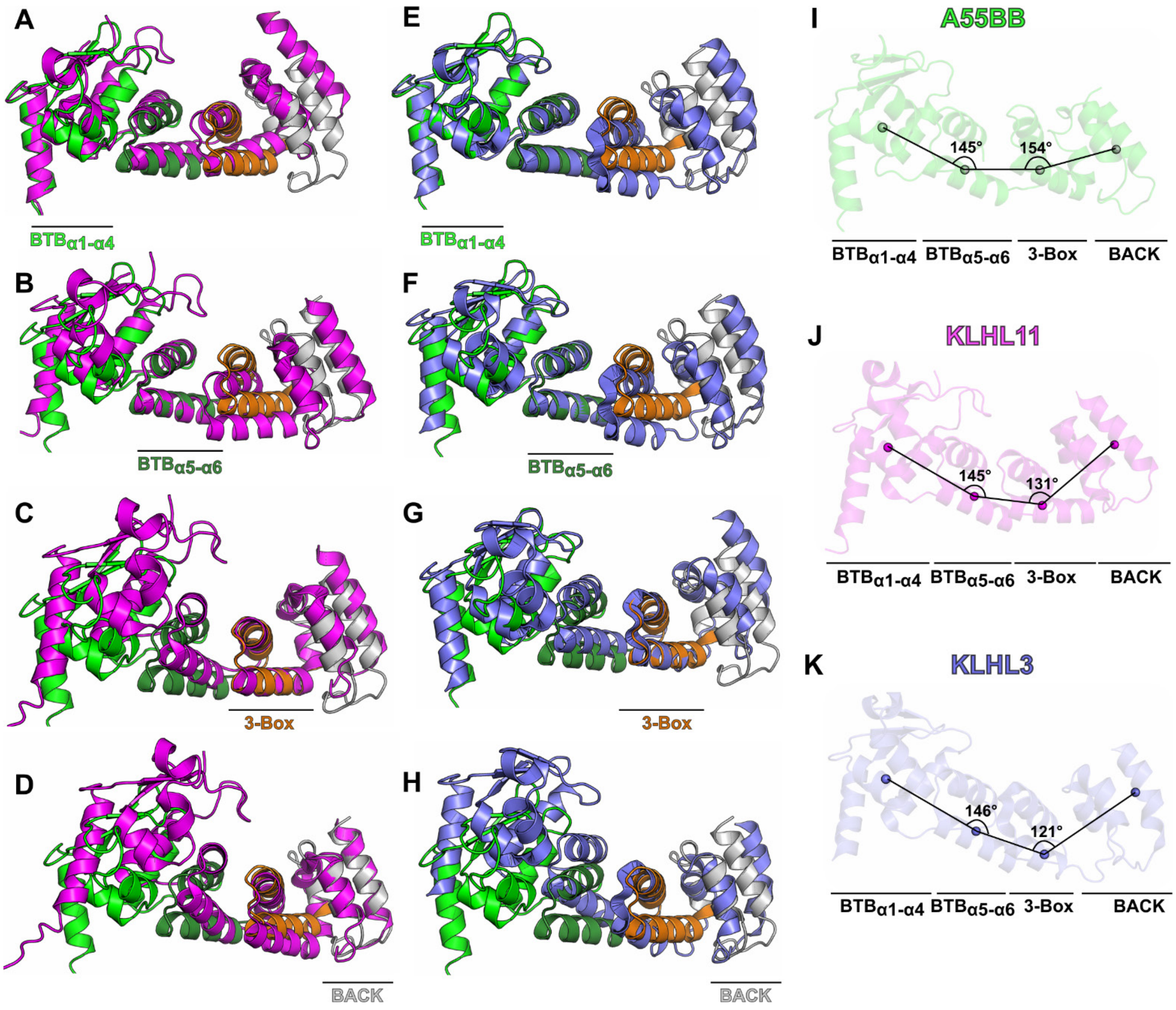

Figure S4. BB proteins exhibit inter-domain flexibility and A55BB domain is more linear than KLHL11 or KLHL3. Structural superposition of KLHL11 (magenta; **A-D**) or KLHL3 (blue; **E-H**) onto A55 $BTB_{\alpha1\text{-}\alpha4}$ (light green), $BTB_{\alpha5\text{-}\alpha6}$ helix-turn-helix (dark green), 3-box region (orange) and BACK domain (grey), respectively. **I-J.** Comparison of inter-domain angles in A55, KLHL11 and KLHL3. The centres of mass (COM) of the subdomains $BTB_{\alpha1\text{-}\alpha4}$, $BTB_{\alpha5\text{-}\alpha6}$, 3-box and BACK in A55 were calculated in PyMOL. The Cα of the residue closest to the COM (COM-Cα) in each subdomain of A55, and the structurally-equivalent Cα atoms in KLHL11 and KLHL3 sub-domains, are shown as spheres. Angles between the COM-Cα atoms of the subdomain $BTB_{\alpha1\text{-}\alpha4}$–$BTB_{\alpha5\text{-}\alpha6}$–3-box and of $BTB_{\alpha5\text{-}\alpha6}$–3-box–BACK are shown. A55 adopts a more linear conformation (wider angle) across the BTB-BACK domain, while both KLHL11 and KLHL3 adopt curved conformations.

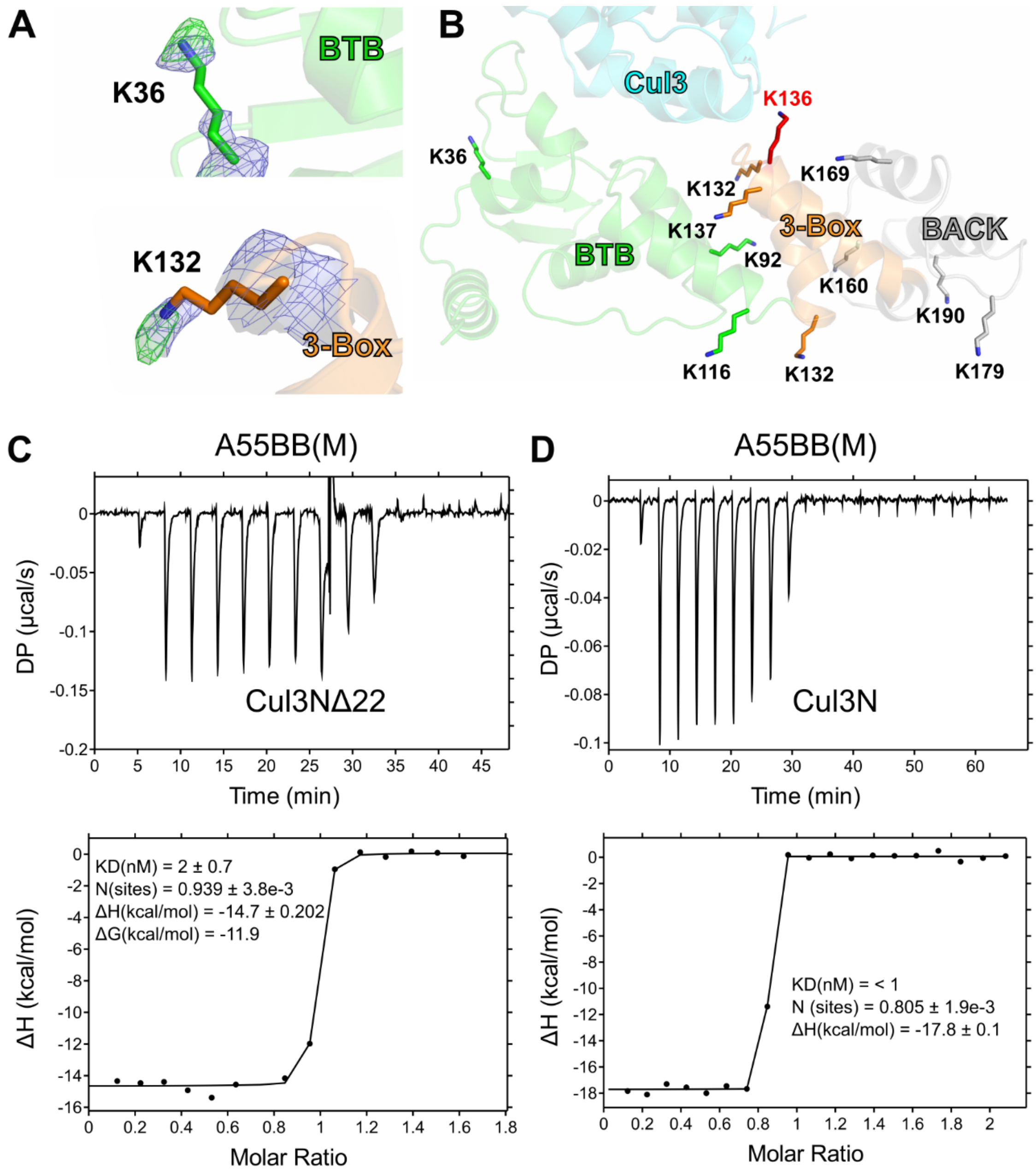

Figure S5. Reductive methylation of A55BB did not disrupt interaction with Cul3. A. $2F_O$-F_C (purple mesh; 1.5σ) and F_O-F_C (green mesh; 3.5σ) maps showing extra density near the side chain amino groups of K36 and K132 in A55 BTB (green) and 3-box (orange) regions, consistent with methylation of these residues. **B.** Side chains of all lysines in A55 are shown as sticks. Only one lysine, K136 (red), is found adjacent to the A55/Cul3 interface. Cul3, BTB, 3-box and BACK domains are in cyan, green, orange and grey, respectively. **C & D.** Representative ITC titration curves showing that A55BB(M) binds to Cul3NΔ22 and Cul3N, respectively, with similar affinities to unmodified A55BB (Fig. 1, *A* and *B*).

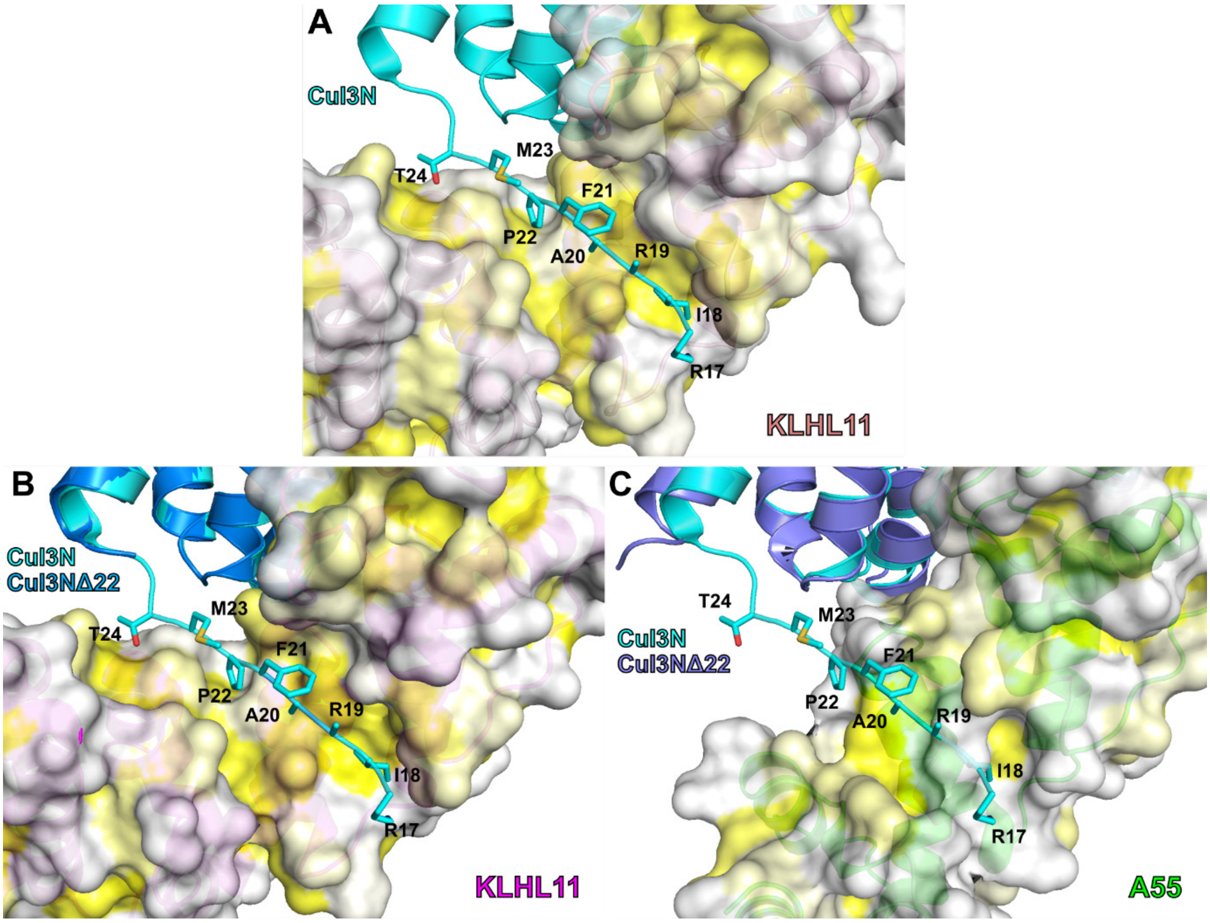

Figure S6. The binding site for Cul3-NTE is not conserved in A55. A. The binding site for Cul3-NTE (residues 17-22) in the structure of KLHL11 (pink) in complex with Cul3N (cyan) (PDB code 4AP2) (3). **B.** The binding site of Cul3-NTE (cyan) from Panel A mapped onto the structure of KLHL11 (magenta) in complex with Cul3NΔ22 (dark blue) (PDB code 4APF) (3). **C.** The Cul3-NTE (cyan) bound to KLHL11 fitted onto the structure of A55BB (green) in complex with Cul3NΔ22 (purple). For Panel A-C, surfaces of KLHL11 and A55BB are shown and colored by residue hydrophobicity from yellow (hydrophobic) to white (polar).

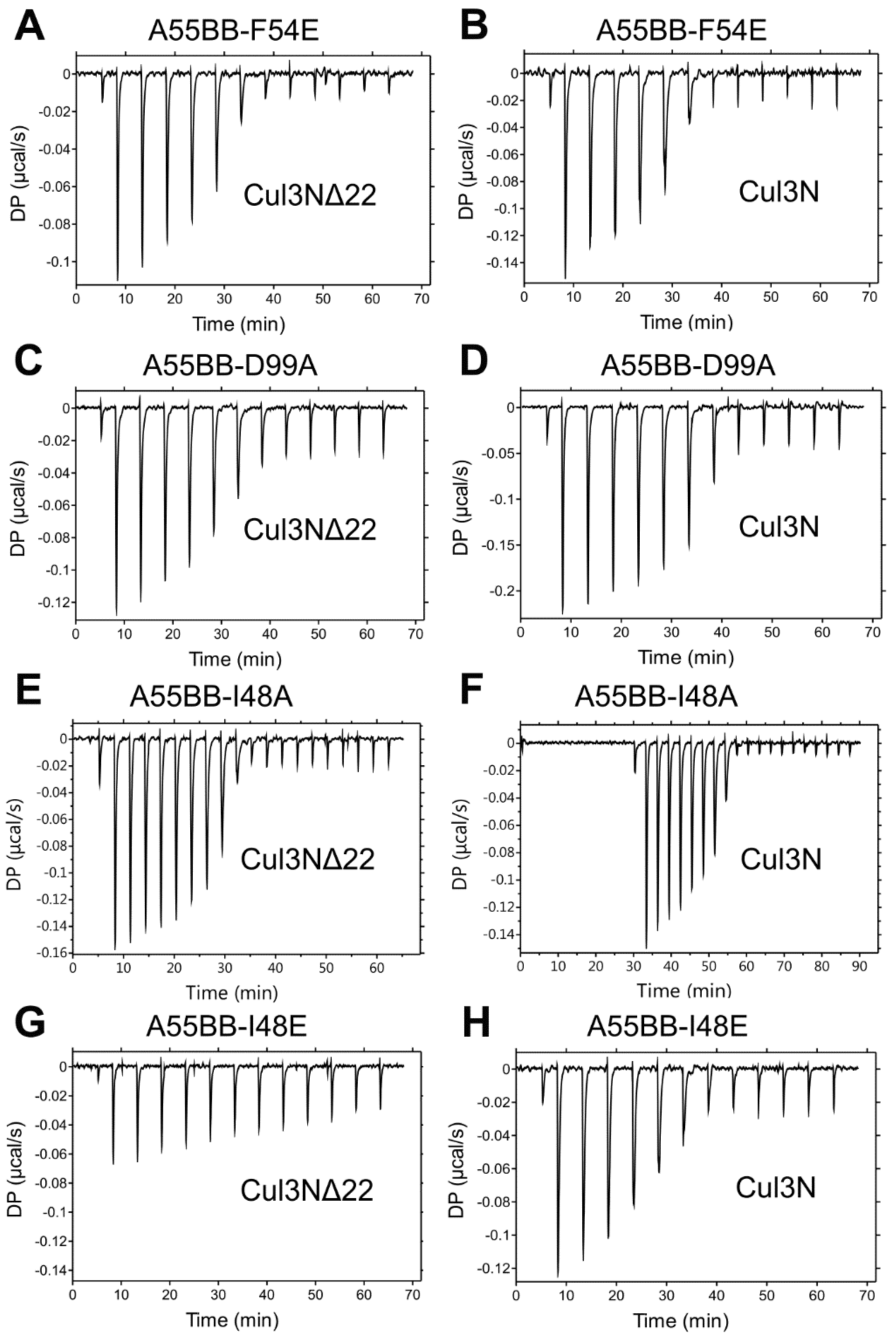

Figure S7. Raw ITC data for titrations of mutant A55BB into Cul3NΔ22 and Cul3N. Differential power (DP) versus time is shown for A55BB-F54E (**A, B**), A55BB-D99A (**C, D**), A55BB-I48A (**E, F**), and A55BB-I48E (**G, H**) binding to Cul3NΔ22 and Cul3N, respectively.

Supplementary References

1. Walter, T. S., Meier, C., Assenberg, R., Au, K. F., Ren, J., Verma, A., Nettleship, J. E., Owens, R. J., Stuart, D. I., and Grimes, J. M. (2006) Lysine methylation as a routine rescue strategy for protein crystallization. *Structure* **14**, 1617-1622
2. Krissinel, E., and Henrick, K. (2007) Inference of macromolecular assemblies from crystalline state. *J Mol Biol* **372**, 774-797
3. Canning, P., Cooper, C. D., Krojer, T., Murray, J. W., Pike, A. C., Chaikuad, A., Keates, T., Thangaratnarajah, C., Hojzan, V., Ayinampudi, V., Marsden, B. D., Gileadi, O., Knapp, S., von Delft, F., and Bullock, A. N. (2013) Structural basis for Cul3 protein assembly with the BTB-Kelch family of E3 ubiquitin ligases. *J Biol Chem* **288**, 7803-7814
4. Errington, W. J., Khan, M. Q., Bueler, S. A., Rubinstein, J. L., Chakrabartty, A., and Prive, G. G. (2012) Adaptor protein self-assembly drives the control of a cullin-RING ubiquitin ligase. *Structure* **20**, 1141-1153
5. Ji, A. X., and Prive, G. G. (2013) Crystal structure of KLHL3 in complex with Cullin3. *PLoS One* **8**, e60445
